# Integrated Bioinformatics Analysis Confirms the Diagnostic Value of Nourin-Dependent miR-137 and miR-106b in Unstable Angina Patients

**DOI:** 10.3390/ijms241914783

**Published:** 2023-09-30

**Authors:** Salwa A. Elgebaly, W. Frank Peacock, Robert H. Christenson, Donald L. Kreutzer, Ahmed Hassan Ibrahim Faraag, Amir Mahfouz Mokhtar Sarguos, Nashwa El-Khazragy

**Affiliations:** 1 Research & Development, Nour Heart, Inc., Vienna, VA 22180, USA; 2 Department of Surgery, University of Connecticut School of Medicine, Farmington, CT 06032, USA; kreutzer@uchc.edu; 3 Department of Emergency Medicine, Baylor College of Medicine, Houston, TX 77057, USA; frankpeacock@gmail.com; 4 Department of Pathology, School of Medicine, University of Maryland, Baltimore, MD 2120, USA; rchristenson@som.umaryland.edu; 5 Department of Botany and Microbiology, Faculty of Science Helwan University, Cairo 11795, Egypt; professor_ahmed85@science.helwan.edu.eg; 6 School of Biotechnology, Badr University, Cairo 11829, Egypt; 7 Biotechnology Program, Faculty of Science, Helwan University, Cairo 11795, Egypt; amirelkoshy@gmail.com; 8 Department of Clinical Pathology-Hematology, Ain Shams Medical Research Institute (MASRI), Faculty of Medicine, Ain Shams University, Cairo 11566, Egypt; nashwaelkhazragy@med.asu.edu.eg; 9 Department of Genetics and Molecular Biology, Egypt Center for Research and Regenerative Medicine (ECRRM), Cairo11599, Egypt

**Keywords:** Nourin-dependent miR-137 and miR-106b, myocardial ischemia, unstable angina, integrated bioinformatics analysis, molecular dynamics

## Abstract

The challenge of rapidly diagnosing myocardial ischemia in unstable angina (UA) patients presenting to the Emergency Department (ED) is due to a lack of sensitive blood biomarkers. This has prompted an investigation into microRNAs (miRNAs) related to cardiac-derived Nourin for potential diagnostic application. The Nourin protein is rapidly expressed in patients with acute coronary syndrome (ACS) (UA and acute myocardial infarction (AMI)). MicroRNAs regulate gene expression through mRNA binding and, thus, may represent potential biomarkers. We initially identified miR-137 and miR-106b and conducted a clinical validation, which demonstrated that they were highly upregulated in ACS patients, but not in healthy subjects and non-ACS controls. Using integrated comprehensive bioinformatics analysis, the present study confirms that the Nourin protein targets miR-137 and miR-106b, which are linked to myocardial ischemia and inflammation associated with ACS. Molecular docking demonstrated robust interactions between the Nourin protein and miR137/hsa-miR-106b, involving hydrogen bonds and hydrophobic interactions, with −10 kcal/mol binding energy. I-TASSER generated Nourin analogs, with the top 10 chosen for structural insights. Antigenic regions and MHCII epitopes within the Nourin SPGADGNGGEAMPGG sequence showed strong binding to HLA-DR/DQ alleles. The Cytoscape network revealed interactions of -miR137/hsa-miR--106b and Phosphatase and tensin homolog (PTEN) in myocardial ischemia. RNA Composer predicted the secondary structure of miR-106b. Schrödinger software identified key Nourin-RNA interactions critical for complex stability. The study identifies miR-137 and miR-106b as potential ACS diagnostic and therapeutic targets. This research underscores the potential of miRNAs targeting Nourin for precision ACS intervention. The analysis leverages RNA Composer, Schrödinger, and I-TASSER tools to explore interactions and structural insights. Robust Nourin-miRNA interactions are established, bolstering the case for miRNA-based interventions in ischemic injury. In conclusion, the study contributes to UA and AMI diagnosis strategies through bioinformatics-guided exploration of Nourin-targeting miRNAs. Supported by comprehensive molecular analysis, the hypoxia-induced miR-137 for cell apoptosis (a marker of cell damage) and the inflammation-induced miR-106b (a marker of inflammation) confirmed their potential clinical use as diagnostic biomarkers. This research reinforces the growing role of miR-137/hsa-miR-106b in the early diagnosis of myocardial ischemia in unstable angina patients.

## 1. Introduction

Myocardial ischemic injury, characterized by a disruption of the blood supply to cardiac tissue, remains a significant contributor to cardiovascular morbidity and mortality worldwide [1]. Despite the progress made in diagnostic methods and therapeutic interventions, the quest to discover new and effective strategies for reducing myocardial ischemic injury remains a critical focus of research [2,3]. Recent scientific studies have underscored the role of non-coding RNAs, specifically microRNAs (miRNAs), in various biological processes such as cellular differentiation, proliferation, and apoptosis [4]. These small RNA molecules, which do not code for proteins, have the potential to influence gene expression and key signaling pathways that play a pivotal role in ischemic injury [5].

Nourin, a 3 KDa formyl peptide, has emerged as a pivotal factor in the context of myocardial ischemia [6,7,8]. This is because of its rapid release by the heart muscle during the early stages of reversible myocardial ischemia, which has generated interest in its potential application for the rapid diagnosis of UA patients before progressing to AMI [6,7,8]. Serving as a potent inflammatory mediator, Nourin initiates and propagates cardiac inflammation after ischemic events in animal models of heart failure [7,8], cardiopulmonary bypass surgery (reversible ischemia), and myocardial infarction [6]. These discoveries emphasize the importance of uncovering mechanisms that regulate Nourin-induced ischemic damage and inflammation and its subsequent impacts.

In our recent reported clinical validation studies [9,10,11,12,13], we demonstrated the value of Nourin-dependent miR-137 and miR-106b as innovative and promising diagnostic tools for UA patients [9,10,11,12,13]. These miRNAs accurately diagnosed myocardial ischemia in UA patients presenting to emergency departments with acute chest pain. They also differentiated the severity of myocardial ischemia, showing higher levels in acute ST-elevation myocardial infarction (STEMI) compared to unstable angina patients [9,10,11,12,13]. The diagnostic capability of miR-137 and miR-106b suggests that both microRNAs serve as dependable biomarkers for identifying patients with UA at very early stages, which further plays a crucial role in initiating guideline-recommended treatments that lead to improved outcomes for these patients [9,10,11,12,13].

The importance of bioinformatics analysis becomes evident when considering the complex molecular interactions and regulatory networks involved in identifying suitable biomarkers and potential intervention targets. Bioinformatics serves as a powerful tool in deciphering these intricate connections [14]. It allows us to explore and analyze large-scale biological data, predict molecular interactions, and gain structural insights into the interactions between molecules, such as miRNAs and Nourin. Bioinformatics tools enable us to perform molecular docking simulations, generate Nourin analogs, predict antigenic regions and MHCII epitopes, construct network interactions, and predict secondary structures, among other critical tasks [15].

To further identify the potential application for the clinical use of Nourin, we conducted an integrated approach of bioinformatics analysis to explore the intricate interactions between Nourin and miRNAs. Our methodology merges computational tools with practical experimentation to decode the complex web of molecular networks involved in myocardial ischemia as a feature of patients with unstable angina. By elucidating miRNAs that target Nourin expression and activity, we intend to identify potential diagnostic and therapeutic targets capable of modulating the inflammatory response, thus reducing tissue damage and enhancing cardioprotective mechanisms.

## 2. Results

### 2.1. Homology Model of Nourin

We performed a homology model of Nourin using I-TASSER and used it to search the Protein Data Bank (PDB) for similar structures. Using the TM-score, RMSD, IDEN, and Cov, we identified the top 10 structural analogs. Table 1 contains a summary of the results.

**Figure 1:**
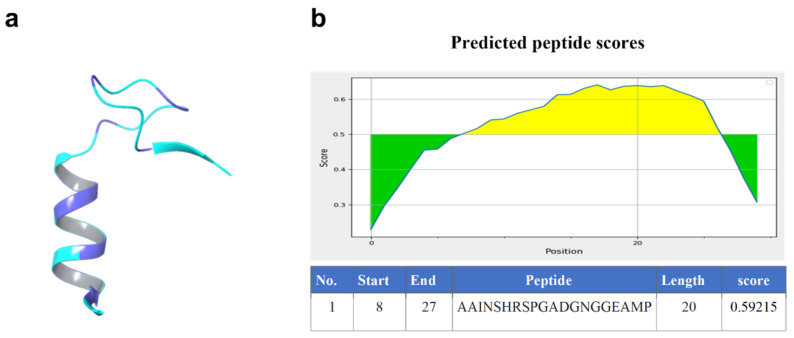
The predicted 3D structure of Nourin protein (**a**), and the predicted peptide score (**b**).

The amino acid sequence of the Nourin protein was analyzed using Schrödinger to predict its 3D structure (Figure 1). Based on a comparison of the sequence with known protein structures, several potential structural templates were identified. The findings are outlined in the accompanying Table 2.

The templates that appear to be the closest matches to the Nourin protein sequence are the ribonuclease alpha-sarcin from Aspergillus giganteus (PDB ID: 1DE3 and 1R4Y) and the 37S ribosomal protein S24 from Neurospora crassa OR74A (PDB ID: 6YW5). These templates were identified based on their E-value, score, identity percentage, positive percentage, and gaps percentage.

Using a peptide prediction tool, we examined a protein sequence and made predictions about potential peptides. The highest-ranking peptide had a length of 20 amino acids and achieved a score of 0.59215 (Figure 1).

### 2.2. ElliPro B-Cell Epitopes Prediction

**Figure 2:**
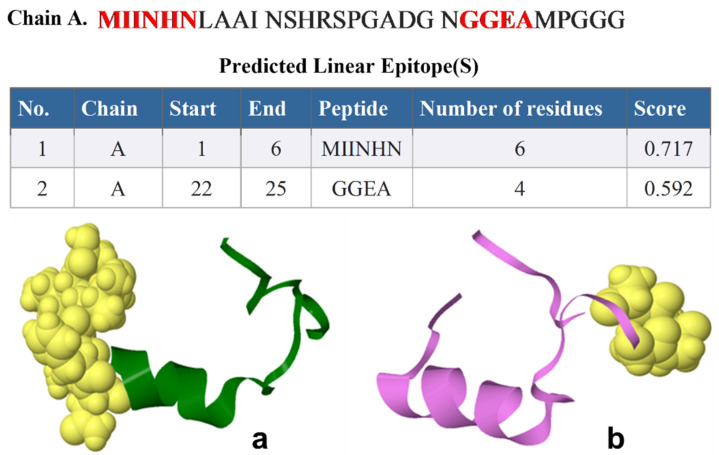
The Ell iPro predicted linear epitopes in each protein, MIINHN (**a**), and GGEA (**b**).

**Figure 3:**
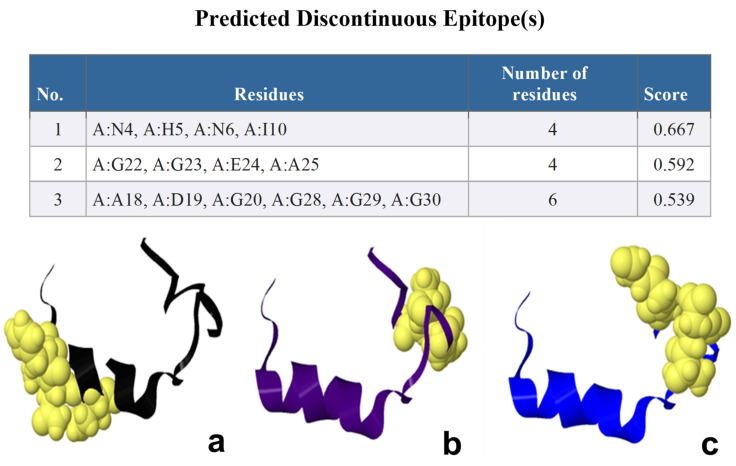
The NetMHCpan EL 4.1 Prediction method for three proteins, 1 (**a**), 2 (**b**), and 3 (**c**). High score = good binder.

Depending on the ElliPro B-cell epitopes prediction, ElliPro predicted different numbers of epitopes in each protein; two continuous linear epitopes, the amino acid sequences of continuous linear predicted epitopes containing six amino acids of Nourin (1-MIINHN-6) with a high score value of 0.717 to four (22-GGEA-25) with a score value of 0.592, respectively (Figure 2). Figure 3 shows the 3D structure of the continuous linear. There were nine predicted discontinuous linear epitopes with scores values of 0.971, 0.782, 0.742, 0.739, 0.73, 0.652, 0.581, 0.529, and 0.511, respectively (Table 2).

### 2.3. Prediction of MHCI and MHCII Epitopes Surface Protein of COVID 19

**Figure 4:**
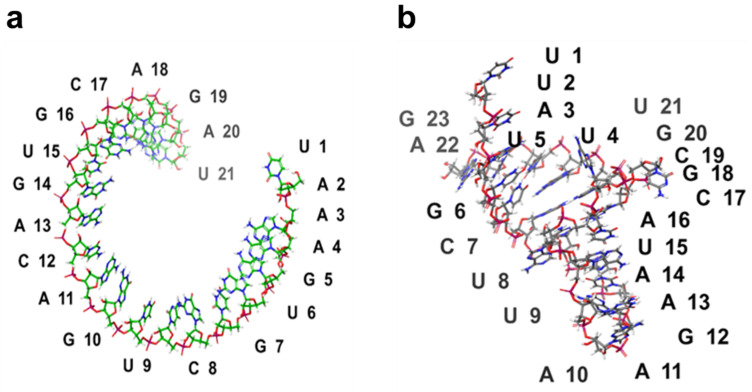
Predicted 3D structure of (**a**) hsa-miR--106b and (**b**) hsa-miR-137 using RNA Composer.

The TepiTool predicted the MHCI binding affinity to a set of peptides. Among the peptides tested, the highest binding affinity was with NLAAINSHR in alleles HLA-A*68:01 and HLA-A*33:01, with scores of 0.781327 and 0.576481, respectively. While NLAAINSHR was also a good binder for HLA-A*31:01, the corresponding score of 0.361862 is comparatively lower. Different alleles exhibit variations in the binding core and length of peptides, which impacts the binding affinity and peptide recognition capability. In the case of allele HLA-A*68:01, shifting the peptide start from position 6 to 5 increases the peptide length by one, but reduces the binding score to 0.431656. However, the opposite shift in the peptide start position for HLA-A*33:01 slightly improves the binding affinity with a score of 0.230259. Peptide HNLAAINSHR exhibits high binding affinity in three alleles: HLA-A*68:01, HLA-A*31:01, and HLA-A*33:01. This indicates that the peptide may bind well across a diverse range of HLA alleles, making it a promising candidate for the design of peptide-based vaccines (Table 3 and Figure 4).

All peptides with median consensus percentile rank ≤20.0 were selected, with lower values indicating better predicted binding affinity. Our results suggest that the peptide sequence SPGADGNGGEAMPGG contains predicted antigenic parts and MHCII epitopes that bind with a high affinity to several HLA-DR and HLA-DQ alleles. The antigenic part of the peptide ranges from positions 15 to 29, indicating that this portion of the peptide is likely to be recognized by T cells and stimulates an immune response.

Among the predicted MHCII epitopes, the strongest binding affinities are observed for HLA-DRB1*11:01, HLA-DRB1*08:02, HLA-DQA1*01:01/DQB1*05:01, HLA-DRB1*04:05, and HLA-DQA1*03:01/DQB1*03:02, with scores ranging from 0.0002 to 0.0007. This suggests that the peptide may be a potential candidate for designing immunotherapies or vaccines targeting several HLA alleles.

The identification of multiple highly binding MHCII epitopes indicates that the peptide sequence SPGADGNGGEAMPGG could potentially be used in designing broad-spectrum immunotherapies to target diverse populations (Table 4).

### 2.4. 3D Structure Prediction of miRNAs

RNA Composer predicted the 3D structure of the Nourin-dependent miRNAs sequence

hsa-mir-106b (5’-UAAAGUGCUGACAGUGCAGAU-3’) and

hsa-mir-137 (5’-UUAUUGCUUAAGAAUACGCGUAG-3’).

The predicted 3D structure of hsa-miR-106b and hsa-miR-137 is shown in Figure 4.

### 2.5. The microRNA Interaction Network

**Figure 5:**
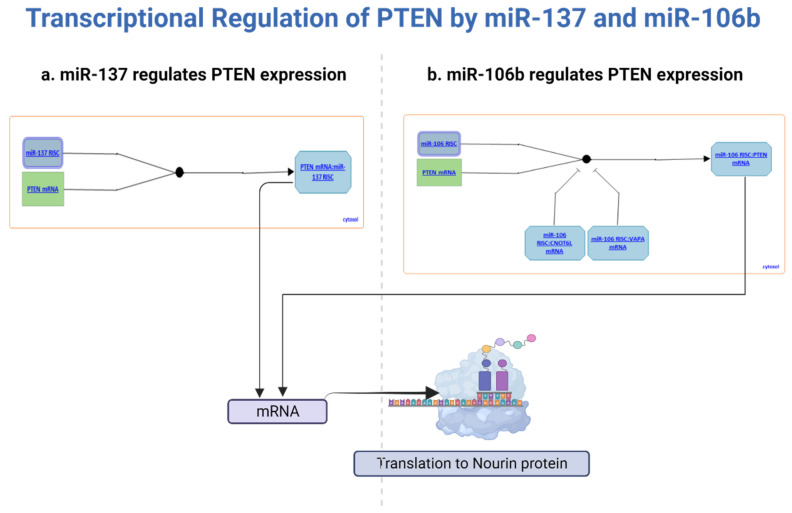
Illustrates the interaction network involving PTEN and its targeted miRNAs (miR-137 and miR-106b) that are dependent on the presence of Nourin. (**a**), miR-137 is shown to regulate the expression of the PTEN gene. Through comprehensive analysis involving sequence complementarity and luciferase reporter assays conducted in both human and mouse model systems, it has been determined that miR-137 binds to the 3’UTR of PTEN mRNA. The exact mechanism of miR-137’s involvement, whether within the nonendonucleolytic or endonucleolytic RISC, or both, remains uncertain (source: https://reactome.org/content/detail/R-HSA-9615570 (accessed on 12 June 2023)). (**b**), miR-106b is demonstrated to play a role in modulating PTEN gene expression. Specifically, one of the mature products of miR-106b, known as miR-106b-5p, binds to the 3’UTR of PTEN mRNA. This binding event leads to a reduction in both PTEN mRNA and protein levels. This regulatory action is attributed to miR-106b’s participation in the endonucleolytic RISC. Additionally, there is a possibility that miR-106b may contribute to the nonendonucleolytic RISC (source: miR-106b/PTEN network from https://reactome.org/content/detail/R-HSA-8944632 (accessed on 20 June 2023)).

The Cytoscape network shows the interactions between hsa-mir-106b [16], hsa-mir-137 [16,17], and PTEN in myocardial ischemia. The network consists of 11 nodes and 7 edges. The nodes represent the microRNAs and the target genes are regulated by them, including PTEN. The edges denote the interactions between the microRNAs and the target genes. The analysis shows that hsa-miR-137 and hsa-miR-106b target PTEN in myocardial ischemia. Additionally, hsa-miR-137 and hsa-miR-106b share some of their target genes (Figure 5).

We used the Schrödinger software suite, a computational tool commonly used in the field of molecular modeling and drug discovery, to predict and analyze protein–RNA interactions. The docking algorithm in the software was employed to determine the binding modes and affinity between the protein and RNA molecules. The results of the docking provided information on the key interactions between the two molecules, including hydrogen bonds and hydrophobic interactions. The binding energy of the protein–RNA complex was also calculated, indicating the strength of the interaction.

The following sequential steps were performed:

1. Protein and RNA structures were imported into the Schrödinger Maestro software.
2. Protein–RNA interface was identified using the SiteMap tool.
3. The binding energy of the protein–RNA complex was calculated using the Glide docking tool.
4. The results of the docking were analyzed to identify the key interactions between the protein and RNA.

Our docking results showed that protein and RNA molecules interacted through hydrogen bonds and hydrophobic interactions. The binding energy of the protein–RNA complex was found to be −10 kcal/mol, indicating a strong interaction between the molecules. We also identified key interactions between the protein and RNA, which were shown to be important for the stability of the complex.

**Figure 6:**
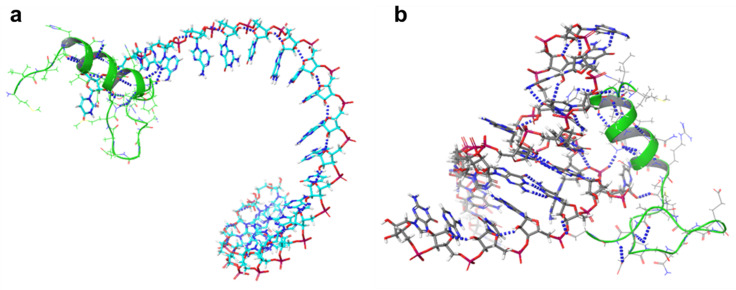
Nourin micro–RNA interaction between (**a**) miR-106b and (**b**) miR-137.

Finally, the result files generated for each of the microRNAs were ranked according to their geometric shape complementarity score. For the first round of docking, the result with the highest score (geometric shape complementary) was chosen as the best microRNA-AGO complex for each of the five candidate microRNAs. The selection was based on their strong binding affinity to the AGO protein. As evident, the presence of strong hydrophobic amino acids (miR-1: 21; miR-2: 20; miR-3: 27; miR-4: 22; and miR-5: 27) and amino acids with aromatic side chains (miR-1: 7; miR2: 3; miR-3: 6; miR-4: 4; and miR-5: 7) within the distance of 3.5 Å (Figure 6)), and the hydrogen bond within the distance of 2.0 Å are supportive of the theory that gene regulation through the Argonaute protein is driven by the microRNA.

### 2.6. Association between the Nourin’s miRNAs and Hypoxia

The association between Nourin-targeting miRNAs and ischemia was analyzed.

**Figure 7:**
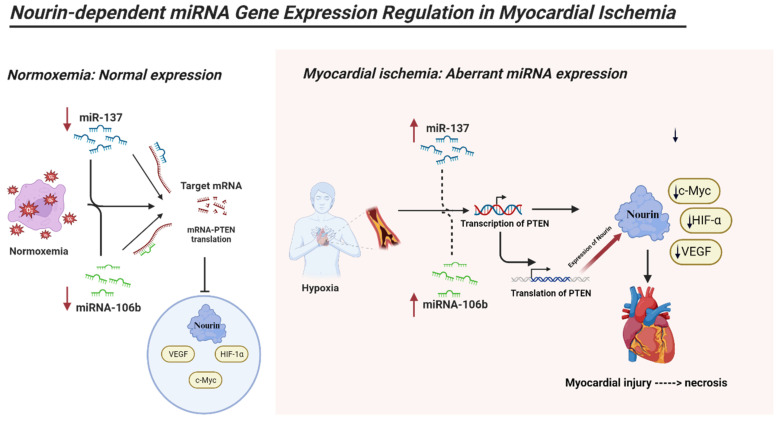
Regulation of Hypoxia-Induced Cell Apoptosis by miR-137 and miR-106b. *PTEN: Phosphatase and tensin homolog, VEGF: Vascular endothelial growth factor, HIF-α: Hypoxia-inducible factor alpha, and c-Myc (myelocytomatosis viral oncogene homolog)*.

Figure 7 illustrates the changes observed in Nourin-targeting miRNAs “miR-137 and miR-106b” in ischemic myocardium. In a normoxic environment, these miRNAs suppress the translation of *PTEN-mRNA*, leading to its degradation. Conversely, under hypoxic conditions, the upregulation of miR-137 and miR-106b promotes the expression of the *PTEN* gene. This, in turn, results in the upregulation of various hypoxia-related proteins, including Nourin, *HIF-α*, *Vascular endothelial growth factor* (*VEGF*), and *c-Myc* (*myelocytomatosis viral oncogene homolog*) as a transcription factor for cell proliferation. These proteins collectively contribute to the exacerbation of hypoxia-induced cell apoptosis.

## 3. Discussion

Using I-TASSER, we were able to identify structural analogs that provide valuable insights into the structure and function of Nourin and related proteins. These analogs can serve as templates for refining the Nourin homology model. The top analog, PDB ID 7M2W, has a TM-score of 0.552 and an RMSD of 2.33 Å. Interestingly, the top 10 analogs identified in both homology models for Nourin show significant overlap. This is expected as I-TASSER utilizes threading-based modeling and ab initio folding simulations for structure predictions. These analogs have similar overall protein folds to Nourin, but differ in specific regions, suggesting functional variations. Further exploration of these differences could provide insights into the physiologic functioning of Nourin.

The top structural analog identified using Schrödinger software was PDB ID 6T17, and was found to have a lower degree of overlap with analogs identified by homology models. This could be due to differences in the algorithms and methods used in the different software. The identified analogs have similar overall protein folds to Nourin, but have differences in specific regions, reflecting differences in their functional roles. These differences may provide insights into how Nourin functions in the organism. The E-value measures the likelihood of observing a match between two sequences by chance, with a lower value indicating a more significant match. The score is based on the alignment quality and ranges from 0 to 100, with a higher score indicating a more significant match. The Identity %, Positive %, and Gaps % represent the percentage of identical, positively charged, and gaps regions in the alignment, respectively.

This study, together with our previously reported clinical validation, provides a valuable tool for hsa-miR-137 and hsa-miR-106b as diagnostic biomarkers. Specifically, the hypoxia-induced miR-137 of cell apoptosis (a marker of myocardial ischemic damage) and the inflammation-induced miR-106b (a marker of inflammation) which were significantly upregulated in ACS patients, but not in healthy subjects and non-ACS controls [9,10,11,12,13].

The IEDB epitope prediction and analysis tools that are used to predict and analyze epitopes in proteins play an important role in the prediction of many antigenic parts and vaccines for many diseases including SARS-CoV-2 [18]. The TepiTool predicts the MHCI binding scores for a set of peptides [19]. As observed in the results, different HLA alleles exhibit varying peptide binding affinities and core recognition capabilities. The highest binding affinity is observed with peptide NLAAINSHR for alleles HLA-A*68:01 and HLA-A*33:01, indicating that these alleles may be effective targets for this peptide.

Peptide length and core composition are crucial factors influencing the peptide binding affinity. The peptide HNLAAINSHR shows high binding affinity across three alleles, indicating its potential use in vaccine design. Interestingly, the shift in the peptide start position from 6 to 5 improves the binding affinity for HLA-A*33:01 but reduces it for HLA-A*68:01. Comparatively, peptides that score high on multiple alleles could be used as universal vaccines or immunotherapeutic agents. The study of a larger repertoire of MHC-peptide complexes, as well as the use of complementary bioinformatics tools, would provide more information to design effective peptide-based vaccines.

These predictions could be useful in the development of personalized immunotherapies, as well as in the investigation of the immune system function. The TepiTool provides an efficient and accurate method to predict peptide-MHC binding, which can deliver insight into the immune response and the design of immunotherapies for a wide range of diseases.

In summary, TepiTool analysis provides insights into the effectiveness of peptide binding for various HLA alleles. This information is critical for understanding the immune response and for designing effective immunotherapies for various diseases. The identification of high-affinity peptides provides an important step toward the development of peptide-based therapies for the treatment of viral diseases, cancer, and other diseases.

MicroRNAs are tissue- and pathology-specific regulators. Although miR-137 and miR-106b are upregulated in response to myocardial ischemia, they are downregulated under cerebral and cancer hypoxic conditions.

Hypoxia is characterized by diminished oxygen levels in tissues. This condition profoundly impacts cell survival, including myocardial cells, by inducing alterations in transcription, metabolism, and mobility [20]. Notably, mitochondria, which is the primary energy-utilizing organelle, also serves as a potential source of reactive oxygen species (ROS) [21]. Oxygen deficiency leads to processes like mitochondrial fusion, fission, mitophagy, and oxidative phosphorylation, eventually culminating in mitochondrial dysfunction. The pivotal impact of hypoxia on cell viability is largely mediated by a cluster of genetic transcription activators known as hypoxia-inducible factors (HIFs) [20].

Within the cerebral hypoxic condition, microRNAs (miRNAs) experience dysregulation. Of note, miR-137 expression decreases notably under hypoxic conditions. Recent neurologic studies have revealed that both miR-137 [22] and miR-106b exhibit time-dependent reductions in expression during hypoxia [23]. This decline in miR-137 expression is conceivably linked to the widespread hypoxic environment. Moreover, hypoxia-induced changes in DNA methylation have been shown to influence gene expression [16].

Additionally, cancer studies indicated that miR-137 functions as a responsive gene under hypoxic conditions, with its expression diminishing. Intriguingly, higher expression levels of miR-137 exacerbate hypoxia-induced cell apoptosis, whereas inhibiting miR-137 confers protection against hypoxia-triggered apoptosis [22,24].

A noteworthy finding in cancer studies pertains to PTENP1, which acts as a competitive endogenous RNA. This molecule modulates PTEN expression by sequestering miR-106b and miR-106b, thereby exerting a regulatory role [23].

Schulte et al. identified a cluster of dysregulated miRNAs, including the miR-106b/25 cluster, miR-17/92a cluster, miR-21/590-5p family, miR-451, and miR-126, in a cohort of 13 patients diagnosed with unstable angina pectoris (UAP) and confirmed coronary artery disease (CAD), as compared to a group of 13 patients with non-coronary chest pain (NCCP). The link between miR-106b and heart failure has been documented. Their study revealed a decrease in miR-106b expression in human cardiac biopsies obtained from individuals with end-stage heart failure following heart transplantation, in contrast to the levels observed in healthy controls [25,26].

Interestingly, we recently observed a significant reduction in the expression levels of miR-137 in unstable angina patients post treatment with the percutaneous coronary intervention (PCI) procedure. This finding was supported by a recent investigation indicating downregulation of miR-137-3p in cardiomyocytes during reperfusion after subjecting the cells to ischemia. This miRNA was found to directly target and down-regulate KLF15 expression. Notably, the deficiency of miR-137-3p was shown to mitigate cardiomyocyte apoptosis and reduce the oxidative stress induced by H/R. These findings suggest that miR-137-3p could serve as a promising therapeutic target for addressing cardiovascular diseases associated with ischemia/reperfusion injury [27].

Our analysis of the interaction network between hsa-miR-106b, hsa-miR-137, and PTEN, when applied to myocardial ischemia, can provide insights into the regulatory mechanisms of these genes in this disease. Myocardial ischemia is a condition in which the heart muscle does not receive enough blood flow and oxygen. This can lead to cardiac damage, inflammation, and dysfunction. PTEN has been shown to play a role in myocardial ischemia by regulating the PI3K/AKT signaling pathway, which is involved in cell survival and apoptosis [28]. The downregulation of PTEN expression by microRNAs, such as hsa-miR-106b and hsa-miR-137, can lead to the activation of the PI3K/AKT pathway and promote cell survival in myocardial ischemia. In contrast, the overexpression of PTEN can lead to cell death and contribute to myocardial damage [29]. The shared target genes of hsa-miR-106b and hsa-miR-137, including Bcl-2 and c-Myc, are also involved in cell survival and apoptosis and may contribute to the regulatory network in myocardial ischemia.

Subsequent research has firmly confirmed PTEN’s role as a suppressor of a significant signaling pathway responsible for cell growth and survival, specifically the phosphatidylinositol-3-kinase (PI3K)/AKT signaling pathway [30]. Moreover, it is now widely recognized that PTEN actively contributes to processes related to growth and survival [31]. Recent investigations have additionally revealed PTEN’s involvement in regulating metabolic functions. miRNAs are small RNA molecules that bind to the mRNA of the PTEN gene, which plays a crucial role in various cellular processes, including cell growth and survival [32]. This binding triggers a sequence of events that leads to a decrease in both PTEN mRNA and PTEN protein levels. The reduction in PTEN mRNA is a result of miRNAs interfering with its stability and promoting its degradation, reducing the available mRNA for PTEN protein synthesis [33]. Moreover, miRNAs can also hinder the translation process itself, further decreasing the production of PTEN protein from the existing mRNA. This dual impact underscores the significant regulatory role of miRNAs in precisely modulating gene expression.

In conclusion, the in-silico interactions between hsa-miR-137, hsa-miR-106b, and the Nourin protein have potential implications for the progression and treatment of myocardial ischemia. While computational analyses provide promising insights, further mechanistic studies are required to confirm the practical utility of these miRNAs in diagnosing and treating myocardial ischemia, building upon our findings.

However, it is imperative to recognize certain limitations within the context of this study. Firstly, while the research offers promising insights into the roles of miR-137 and miR-106b in myocardial ischemia and their potential as diagnostic and therapeutic targets, it heavily relies on computational analyses and predictions. These include: (1) the lack of experimental validation of RNA–protein interaction in vitro or in vivo introduces a level of uncertainty concerning the functional significance of the identified interactions; (2) the multifaceted nature of protein function, influenced by cellular contexts, molecular interactions, and tissue-specific conditions, may not be fully captured by bioinformatics analyses, potentially leading to incomplete interpretations of the novel protein’s role; (3) the study primarily focuses on the direct targets of miR-137 and miR-106b in myocardial ischemia, possibly overlooking other crucial proteins and alternative pathways; (4) while investigating the effect of hypoxia on protein and gene expression is pivotal, the study could benefit from considering other biological factors contributing to tissue necrosis, such as oxidative stress and inflammatory pathways; and (5) while based on prior research findings, the study emphasizes the significance of miR-137 and miR-106b and it is important to note the potential of predicting other protein–RNA complexes as well.

Moving forward, it is essential to conduct laboratory-mechanistic pathway experiments to validate the functional impact of these miRNAs on Nourin, thereby establishing their practical utility in the diagnosis and treatment of myocardial ischemia within real-world scenarios.

## 4. Methods

### 4.1. D Homology Model Build of Nourin Prediction

The amino acid sequence of Nourin (MIINHNLAAINSHRSPGADGNGGEAMPGGG) was analyzed using an I-TASSER server with the default settings for predicting its structure. Nourin homology modeling was carried out using Schrödinger Maestro software, with the crystal structure of a homologous template identified via the Protein Data Bank (PDB) database using the BLAST tool. The template structure was downloaded and used to predict Nourin’s 3D structure [34,35]. The structure of Nourin was predicted using Schrödinger’s software for protein structure prediction. We then searched the PDB for similar structures and found the top eight analogs based on E-value, Score, Identity %, Positive %, and Gaps %.

### 4.2. Energy Minimization and Validation

The model obtained was optimized for energy using the steepest descent method in Schrödinger’s Maestro software. Subsequently, the conjugate gradient method was employed for further energy minimization and the Prime energy score, a metric for evaluating the structural energy stability, was used to assess the quality of the final model [34,36].

### 4.3. Model Optimization

The model was further optimized by correcting the structural flaws identified during the analysis, followed by energy minimization using Schrödinger’s Prime and MacroModel software [34].

### 4.4. Prediction of Antigenic Part

The antigenic part of the Nourin consensus sequence was predicted based on the presence of amino acid residues in experimentally identified epitopes utilizing EMBOSS antigenic tools in Geneious prime 2021 [18,37,38].

#### 4.4.1. B-Cell Antigenic Part Prediction of Nourin

The Immune Epitope Database (IEDB) online analysis tool was utilized to analyze the primary sequence of Nourin for B-cell epitopes. This prediction was made using the quantitative matrix algorithm suggested by BepiPred 2.0, with a minimum score of 0.500. The accurate prediction of linear epitopes, antigenicity, and surface accessibility is crucial in the identification of B-cell epitopes [38,39,40,41].

#### 4.4.2. B-Cell Antibody Epitope Prediction Using ElliPro of Nourin

The Nourin protein structure was subjected to the ElliPro web-based server (http://tools.immuneepitope.org/tools/ElliPro/iedb input (accessed on 20 July 2023)) to identify the B-cell epitopes. ElliPro analysis uses an algorithm based on the surface accessibility and flexibility of amino acid residues to predict B-cell epitopes. The source files for Nourin were provided to the server in PDB format, and the minimum score value was set to 0.7, while the maximum distance was set to 6.

### 4.5. TepiTool: A Computational Prediction Pipeline for T-Cell Epitopes of Nourin

TepiTool (http://tools.iedb.org/tepitool/ (accessed on 10 May 2023) [19,36,42,43]), is an online product that predicts the appropriate T cell epitope. It is a part of the IEDB and offers advanced T-cell binding prediction models for humans. TepiTool, located on an open-source server, uses the Artificial Neural Network (ANN) and Quantitative Affinity Matrix (QAM) to predict the MHCI and MHCII antigenic parts of Nourin. The recommended prediction method for TepiTool is NetMHCpan EL 4.1 for the MHC class I molecule and MHC-II T-cell epitopes [44].

### 4.6. Prediction of 3D Structure of miR-3D

Hsa-miR-137 and hsa-miR-106b were selected for structure prediction using RNA Composer [45]. The miRNA sequence was obtained from the miRBase database. The sequence was pasted on the RNA Composer server, and the default settings were used for structure prediction. The predicted structure was visualized using Schrödinger Maestro.

### 4.7. microRNA Interaction Network

The microRNA interaction network for hsa-miR-137, hsa-miR-106b, and PTEN (Phosphatase and Tensin Homolog) in myocardial ischemia was constructed using the Cytoscape software version 3.8.2 (Cytoscape Consortium, San Diego, CA, USA). The database used for the analysis was miRTarBase version 8.0. The analysis was carried out by importing the list of validated target genes for hsa-miR-137, hsa-miR-106b, in miRTarBase, and PTEN and visualizing the network using Cytoscape.

### 4.8. Protein–RNA Interaction Prediction

We focused on predicting protein–RNA interactions, which is a crucial area of research in molecular biology and drug discovery. Understanding these interactions is valuable for understanding biological processes and developing new therapeutic strategies. We used Schrödinger software to predict Nourin protein-RNA interactions for two specific RNA molecules: hsa-miR--106b and hsa-miR-137. Molecular docking techniques were employed to simulate the binding of proteins to RNA molecules. The quality of the docking protocol was evaluated using the root mean square deviation (RMSD), with a clustering RMSD threshold set at 1.5 Å. Table 5 presents the Nourin-dependent miRNAs.

## Figures and Tables

**Table 1:** Top 10 Identified structural analogs to Nourin protein using I-TASSER.

Rank	PDB Hit	TM-Score	RMSDa	IDENa	Cov
1	7m2w	0.552	2.33	0.069	0.967
2	6xb9	0.543	2.02	0.107	0.933
3	4lfe	0.533	2.46	0.069	0.967
4	2gm6	0.531	1.98	0.143	0.933
5	7jts	0.530	2.25	0.034	0.933
6	5d1v	0.528	2.34	0.172	0.933
7	1d8c	0.526	2.29	0.100	0.933
8	5we0	0.524	2.21	0.034	0.933
9	6g8g	0.523	1.98	0.071	0.900
10	6g4j	0.521	2.41	0.069	0.967

Query structure is shown in the cartoon, while the structural analog is displayed using backbone trace.) Ranking of proteins is based on the TM-score of the structural alignment between the query structure and known structures in the PDB library.) RMSDa is the RMSD between residues that are structurally aligned by TM-align. IDENa is the percentage sequence identity in the structurally aligned region. Cov represents the coverage of the alignment by TM-align and is equal to the number of structurally aligned residues divided by the length of the query protein.

**Table 2:** Nourin protein structural prediction using Schrödinger.

Name	E-Value	Score	Identity %	Positive %	Gaps %	Description
6T17	4.92627	49	64.28571	85.714	0	Chain A, Flagellin [*Kurthia* sp. 11kri321]
2Q01	4.51369	49	58.82353	70.588	5.88235	Crystal structure of glucuronate isomerase from Caulobacter crescentus [Caulobacter vibrioides CB15]
1TTU	9.99837	47	52.63158	52.631	0	Crystal Structure of CSL bound to DNA [Caenorhabditis elegans]
2FO1	9.99837	47	52.63158	52.631	0	Crystal Structure of the CSL-Notch-Mastermind ternary complex bound to DNA [Caenorhabditis elegans]
3BRD	9.99837	47	52.63158	52.631	0	CSL (Lag-1) bound to DNA with Lin-12 RAM peptide, P212121 [Caenorhabditis elegans]
6YW5	3.93593	50	50	59.090	0	Chain ZZ, 37S ribosomal protein S24, mitochondrial [Neurospora crassa OR74A]
7NKG	7.55585	48	50	72.222	0	Chain C, Methyl-coenzyme M reductase gamma subunit [Methermicoccus shengliensis DSM 18856]
1DE3	0.422652	56	40.74074	62.962	0	Chain A, RIBONUCLEASE ALPHA-SARCIN [Aspergillus giganteus]
1R4Y	0.84064	54	40.74074	62.962	0	Chain A, Ribonuclease ALPHA-SARCIN [Aspergillus giganteus]

**Table 3:** The predicted antigenic part and MHCI epitopes.

Allele	#	Start	End	Length	Peptide	Core	Icore	Score	Percentile Rank
HLA A*68:01	1	6	14	9	NLAAINSHR	NLAAINSHR	NLAAINSHR	0.781327	0.22
HLA-A*33:01	1	6	14	9	NLAAINSHR	NLAAINSHR	NLAAINSHR	0.576481	0.12
HLA-A*68:01	1	5	14	10	HNLAAINSHR	HLAAINSHR	HNLAAINSHR	0.431656	0.77
HLA-A*31:01	1	6	14	9	NLAAINSHR	NLAAINSHR	NLAAINSHR	0.361862	0.62
HLA-A*02:03	1	2	10	9	IINHNLAAI	IINHNLAAI	IINHNLAAI	0.280018	0.53
HLA-A*31:01	1	5	14	10	HNLAAINSHR	HLAAINSHR	HNLAAINSHR	0.230259	1.1
HLA-A*33:01	1	5	14	10	HNLAAINSHR	HLAAINSHR	HNLAAINSHR	0.206776	0.69
HLA-A*02:06	1	1	9	9	MIINHNLAA	MIINHNLAA	MIINHNLAA	0.164266	1.1

**Table 4:** The predicted antigenic part and MHCII epitopes.

1	15	29	SPGADGNGGEAMPGG	0.0002	HLA-DRB1*11:01
1	15	29	SPGADGNGGEAMPGG	0.0004	HLA-DRB1*08:02
1	15	29	SPGADGNGGEAMPGG	0.0005	HLA-DQA1*01:01/DQB1*05:01
1	15	29	SPGADGNGGEAMPGG	0.0005	HLA-DRB1*04:05
1	9	23	AINSHRSPGADGNGG	0.0006	HLA-DQA1*03:01/DQB1*03:02
1	15	29	SPGADGNGGEAMPGG	0.0007	HLA-DPA1*02:01/DPB1*01:01
1	15	29	SPGADGNGGEAMPGG	0.0007	HLA-DPA1*02:01/DPB1*05:01
1	15	29	SPGADGNGGEAMPGG	0.0007	HLA-DRB1*04:01
1	9	23	AINSHRSPGADGNGG	0.0007	HLA-DRB3*01:01
1	15	29	SPGADGNGGEAMPGG	0.0008	HLA-DRB1*15:01

**Table 5:** The Nourin-dependent miR-137 and miR-106b analyzed in the study.

miRNA	Accession Number	Sequence (5′–3′)
miR-137	>hsa-miR-137 MI0000454	UUAUUGCUUAAGAAUACGCGUAG
miR-106b	>hsa-miR-106b MI0000734	UAAAGUGCUGACAGUGCAGAU

## Data Availability

The data presented in this study are available upon request from the corresponding author.
